# Impact of Preoperative Neuropraxia on Surgical Duration Following Pediatric Supracondylar Fracture of the Humerus: A Retrospective Cohort Study

**DOI:** 10.1055/s-0043-1771012

**Published:** 2023-07-03

**Authors:** Yazeed Alayed, Bander S. Alrashedan, Sultan K. Almisfer, Ali M. Aldossari

**Affiliations:** 1 Department of Pediatrics, Children Hospital, King Saud Medical City, Ulaishah, Riyadh, Saudi Arabia; 2 Department of Orthopedic Surgery, King Saud Medical City, Ulaishah, Riyadh, Saudi Arabia; 3 Division of Paediatric Orthopaedic Surgery, Department of Orthopedic Surgery, King Saud Medical City, Ulaishah, Riyadh, Saudi Arabia

**Keywords:** child, supracondylar humeral fractures, recovery of function, prognosis, K-wires

## Abstract

**Background** Supracondylar fractures of the humerus (SCFHs) are the most common type of elbow fracture in children. Because of the influence on functional outcome, neuropraxia is one of the most common concerns at presentation. The impact of preoperative neuropraxia on surgery duration is not extensively probed. The clinical implications of several other risk factors associated with preoperative neuropraxia at presentation may contribute to longer surgical duration of SCFH.

**Hypothesis** Preoperative neuropraxia is likely to increase surgery duration in patients who sustained SCFH.

**Patients and Methods** This is a retrospective cohort analysis. Sixty-six patients who sustained surgical pediatric supracondylar humerus fracture were included in the study. Baseline characteristics including age, gender, the type of fracture according to Gartland classification, mechanism of injury, patient weight, side of injury, and associated nerve injury were included in the study. Logistic regression analysis was performed using mean surgery duration as the main dependent variable and age, gender, fracture type according to the mechanism of injury, Gartland classification, injured arm, vascular status, time from presentation to surgery, weight, type of surgery, medial K-wire use, and afterhours surgery as the independent variables. A follow-up of 1 year was implemented.

**Result** The overall preoperative neuropraxia rate was 9.1%. The mean surgery duration was 57.6 ± 5.6 minutes. The mean duration of closed reduction and percutaneous pinning surgeries was 48.5 ± 5.3 minutes, whereas the mean duration of open reduction and internal fixation (ORIF) surgeries was 129.3 ± 15.1 minutes. Preoperative neuropraxia was associated with an overall increase in the surgery duration ( *p* < 0.017). Bivariate binary regression analysis showed a significant correlation between the increase of surgery duration and flexion-type fracture (odds ratio = 11, *p* < 0.038) as well as ORIF (odds ratio = 26.2, *p* < 0.001).

**Conclusion** Preoperative neuropraxia and flexion-type fractures convey a potential longer surgical duration in pediatric supracondylar fracture.

**Level of Evidence** Prognostic III.

## Introduction

Supracondylar fractures of the humerus (SCFH) are the most common type of elbow fracture in children. 1 2 3 4 It constitutes approximately 50 to 70% of all fractures confined around the elbow. 3 5 6 SCFH extension-type can be classified into four subtypes according to the degree of fracture displacement. These subtypes are widely known as the Gartland classification (GC); type I is often nondisplaced and require conservative management, whereas types II and III depict various degrees of displacement with intact posterior cortex or complete displacement and instability, respectively. Type IV portrays a marked displacement with no periosteal contact at the fracture site and requires intraoperative diagnosis. 7 8 Notwithstanding the various possible complications associated with SCFH, nerve injury remains among the highest concerns upon presentation due to its impact on functional outcome. Neuropraxia can be secondary to stretching, entrapment, or compression of the nerve at the fracture site causing segmental demyelination leading to transient weakness or paraesthesia. 9 10

Adequate surgical reduction and fixation is paramount for desirable radiographic outcomes and is independent of a specific postoperative immobilization protocol. 11 Thus, identifying prognostic predictors associated with an increased rate of neuropraxia following injury is highly crucial to anticipate outcomes and counsel the family regarding expected short-term complications. 12 13 In addition, selection bias seems to also play a role in the increase of open reduction and internal fixation (ORIF) operations, as many surgeons tend to perform ORIF in patients presenting with severe fracture displacement and depending on the mechanism of injury. 14 Moreover, controversies on the necessity of emergency surgical interventions performed at night for patients with nerve injury to ensure better functional outcome yielded no consensus. 15 16 17

The purpose of the study was to evaluate mainly the impact of preoperative neuropraxia on surgery duration. We hypothesized that preoperative neuropraxia is likely to increase the surgery duration. In addition, emergency intervention and severe fracture types are potentially associated with an increase in operation duration as well. Identifying these factors may help surgeons anticipate the sequela of such injury.

## Patients and Methods

### Patients

This is a single-center retrospective cohort study of patients up to 14 years of age. Inquiry about the number of surgical operations performed under the orthopaedic surgery department from 2016 until 2020 yielded a total of 1,530 patients. Of these, 66 patients who sustained a surgical pediatric SCFH GC II, III, and IV were included. Forty other patients were excluded from the study for the following reasons: (1) different fracture sites such as lateral condylar fracture; (2) age more than 14 years; (3) concurrent lateral condylar fracture of the humerus; and (4) loss to follow-up or lack of adequate operative data and imaging. Patients were followed up for up to 1 year postoperatively in an outpatient department.

### Methods

Operative data such as time of the injury, time of operation (working or afterhours operations), duration of operation, method of reduction (closed or open), side of K-wires used, and vascular status were noted as well. Patients were allocated into two different groups of either more or less than 1 hour of surgery duration. Physical examination notes written by the orthopaedic team upon admission regarding neurovascular status were recorded as pink and pulsating or pulseless pink or pale and pulseless compared with noninjured extremity. Fracture classification according to the GC was reported based on radiographs obtained preoperatively and intraoperatively. All surgeries were performed by board-certified orthopaedic surgeons with a direct or remote supervision of certified pediatric orthopaedic surgeons. Postoperative immobilization prior to wakening the patient from anesthesia consisted of a well-padded above-elbow backslab applied at around 100 degrees of elbow flexion. The cast is typically discontinued within 3 to 4 weeks from surgery with in-office K-wires removal. Neuropraxia rate was defined as any temporary loss of sensation or weakness in either radial, anterior interosseous of the median, or ulnar nerves.

### Statistical Analysis

Chi-square tests were used to predict the association between exposure risk factors and outcome variables. Independent sample *t* -test was used to compare means of independent variables that are normally distributed. Mann–Whitney *U* test was used to compare between continuous outcome variable and exposure factors that are not normally distributed. Binary logistic regression analysis was performed using preoperative neuropraxia cases as the dependent variable and gender, age, injured arm, weight, fracture type according to the mechanism of injury, time of surgery (working hours or off-duty hours), time from presentation to surgery, GC, and surgical duration as the independent variables. *p* < 0.05 was considered statistically significant. Odds ratio (OR), 95% confidence interval (CI), and *p* -value were obtained for the independent variables. Data analysis was performed by Statistical Package for the Social Sciences (SPSS) (IBM SPSS Statistics for Windows, Version 25.0. Armonk, NY: IBM Corp.).

## Results

The study included 39 (59.1%) female and 27 (40.9%) male patients. Ages ranged from 1 to 14 years, with a mean age of 5.4 years. The fracture was left-sided in 41 (62.1%) patients and right-sided in 25 (37.9%) patients. There were 25 (37.9%) Gartland type II, 33 (50%) type III, and 3 (4.5%) type IV fractures. Also, 61 (92.4%) were extension-type fractures, while 5 (7.6%) were flexion-type fractures. Fifty-nine (89.4%) patients presented with a pink, warm, and palpable distal pulse as opposed to seven (10.6%) patients, who presented with pink, warm, and pulseless hand ( Table 1 ).

**Table 1:** Baseline characteristics for 66 patients included in the study

Injury pattern	Preoperative neuropraxia ( *n* , %)	Age (mean ± SD)	Male/female	Medial K-wire use ( *n* , %)	Right arm/left arm	Surgery duration (mean ± SD)
**Extension** t **ype**	4 (6.6)	5.2 ± 0.3	26/35	37 (56.0)	22/38	52 ± 5.2
**Flexion** t **ype**	2 (3.3)	6.6 ± 1.6	1/5	5 (7.5)	3/3	125.8 ± 26
**GII**	–	5.6 ± 0.45	9/16	12 (18.1)	7/18	34.6 ± 4.1
**GIII**	2 (3.3)	4.7 ± 0.42	16/17	24 (36.3)	15/18	60.4 ± 6.4
**GIV**	2 (3.3)	7.7 ± 1.7	2/1	1 (1.5)	1/2	140 ± 90

Abbreviations: GII/GIII/GIV, Gartland type II/type III/type IV; SD, standard deviation.

Fifty-four (81.8%) patients treated using closed reduction with percutaneous pinning (CRPP) with K-wires, whereas 12 (18.2%) were treated using ORIF. The mean duration of CRPP surgeries was 48.5 ± 5.3 minutes, while ORIF surgeries had a mean duration of 129.3 ± 15.1 minutes. Of all cases, 4% of Gartland II, 21% of Gartland III, 66.6% of Gartland IV, and 50% of flexion-type fractures needed ORIF. All ORIF cases were initially complicated CRPP requiring conversion.

The overall preoperative neuropraxia rate was 9.1%. Ulnar and anterior interosseous nerve (AIN) nerve palsy rate was reported as 4.5% for each nerve involvement. There was no radial nerve palsy reported. Preoperative AIN neurapraxias were mainly weakness of the affected myotome as opposed to merely sensory involvement in the ulnar nerve distribution. Recovery was complete for all preoperative neuropraxia cases within 6 months of hospital discharge. The prognostic indicators of preoperative neuropraxia are listed in Table 2 .

**Table 2:** Prognostic indicators of preoperative neuropraxia at presentation

Variables	Preoperative neuropraxia ( *N* = 6)	Intact neurological function ( *N* = 60)	*p* -Value
**Age** , m **ean ± SD**	6.8 ± 1.3	5.2 ± 0.3	0.16
**Male/** f **emale** , ***n***	2/4	25/35	0.69
**Extension** t **ype** , ***n*** ( **%)**	4 (66.6)	57 (95)	0.03*
**Flexion** t **ype** , ***n*** ( **%)**	2 (33.3)	3 (5)	0.03*
**GII** , *n* (%)	0	25 (41.6)	0.052
**GIII** , *n* (%)	2 (33.3)	31 (51.7)	0.39
**GIV** , *n* (%)	2 (33.3)	1 (1.6)	0.001*
**Pulseless** p **ink** , *n* (%)	5 (83.3)	2 (3.3)	0.001*

Abbreviations: GII/GIII/GIV, Gartland type II/type III/type IV; SD, standard deviation.

* Statistically significant at 5% level.

The presence of preoperative neuropraxia was associated with an increase in the surgery duration ( *p* < 0.017). When corrected for confounders, patients who had preoperative neuropraxia and underwent CRPP showed no significant difference in surgery duration ( *p* > 0.39) in contrast to patients who underwent ORIF conversion showing a significant difference with the increase of surgical duration ( *p* < 0.018).

Bivariate binary logistic regression analysis of surgery duration of >1 hour showed a significant correlation with flexion-type fracture (OR = 11, *p* < 0.037) and ORIF (OR = 26.2, *p* < 0.001). Regression analyses reveal a significant association of flexion-type fracture and ORIF with surgery duration lasting >1 hour. Surgeries performed during daytime and afterhours did not show a significant correlation with the duration of surgery (OR = 0.48, *p* > 0.38) ( Table 3 ).

**Table 3:** Bivariate binary logistic regression analysis of surgery duration > 1 h with prognostic determinants

Variables	Odds ratio (95% CI)	*p* -Value
**Age**	0.99 (0.97–1.01)	0.37
**Gender (** f **emale)**	1.4 (0.50–4.4)	0.52
**Weight**	0.96 (0.88–1.05)	0.40
**Injured arm (right)**	1.2 (0.4–3.7)	0.75
**Fracture type**		
** Extension**	5.5 (0.91–32.9)	0.037*
** Flexion**	11.0 (1.1–105.9)	0.038*
**Time from** p **resentation to** s **urgery**	0.93 (0.84–1.02)	0.14
**Pink pulseless vascular status**	1.7 (0.35–8.7)	0.49
**Surgery** t **ime**		
** Working day operations**	1.00 (0.34–1.9)	0.9
** Afterhours operations**	0.48 (0.09–2.5)	0.38
**Type of** o **peration**		
** CRPP**	0.2 (0.002–0.16)	0.001
** ORIF**	26.2 (4.9–139.4)	0.001*
**Medial K-wire use**	0.3 (0.09 to 1.06)	0.063

Abbreviations: CI, confidence interval; CRPP, closed reduction and percutaneous pinning; ORIF, open reduction and internal fixation.

* Statistically significant at 5% level.

Longer surgery duration was associated with patients who presented with pink pulseless hand preoperatively ( *p* < 0.02). Another association was found between the presence of preoperative neuropraxia and the increase of surgery duration performed at night ( *p* < 0.032, 95% CI: 0.95–1.00).

## Discussion

Studies on association between surgery duration and potential risk factors are scarce. Our study investigates the association between the previously mentioned factors with surgery duration. The authors assume that preoperative neurapraxia might be a predictor of longer surgical duration. To our knowledge, this is the first study to investigate the impact of multiple potential risk factors on surgery duration.

Our study indicates that there may be a potential association between the presence of preoperative neuropraxia and surgery duration. The presence of preoperative neuropraxia prior to surgical reduction indicates a more likelihood that surgery will take longer. This finding should be interpreted with caution. The difference may be attributed to surgical confounders such as extensive soft-tissue involvement, low fracture line, and fracture comminution. 18 Interestingly, Sun et al 19 found that the rate of open reduction increased from 10.2 to 29.1% once the reduction has been delayed for more than 8 hours. The reason postulated was, due to extensive soft-tissue swelling hindering appropriate palpation of fracture fragment, preoperative neuropraxia is more associated with GIII, GIV, and flexion type. Thus, a provision over the need to avoid ORIF is paramount if soft-tissue swelling is expected.

Soldado et al 20 found that type IV fractures in their series were presented with an intact medial or lateral periosteal hinge with associated disruption of near-complete circumference of the periosteum. An explicit technical insight on closed reduction maneuvers of these injuries were demonstrated which are implemented in our practice. In spite of that, closed reduction attempts deemed without success in 80% of type IV fractures amongst patients with neurapraxia at presentation in our study. The likely reason behind having a high rate of conversion to open approach is the concerning associated soft tissue injury. 21

Chukwunyerenwa et al 22 described the technical difficulties encountered in the treatment of flexion-type SCFH. Our study showed similar evidence of treatment difficulty represented by longer surgical duration in flexion-type fractures. Surgeons may extrapolate a difficult reduction if neuropraxia is noted prior to surgery, rendering a more careful approach to site of injury, which eventually results in longer surgery duration.

A significant correlation with the increased rate of overall preoperative neuropraxia is identified in patients who present with pulseless pink hands. Several studies 23 24 25 26 argue that surgical management in patients who sustained SCFH with intact neurovascular status can be safely delayed. In our opinion, the aim of prompt surgical intervention for a patient with severe fracture type and vascular compromise should not target decreasing the incidence of nerve injury as by secondary intention because some cases will have concurrent postoperative neuropraxia regardless of urgent intervention. Surgeons should be aware that patients with preoperative neuropraxia and compromise vascular status are more likely to have longer operation duration.

We advocate being more attentive to children presenting with preoperative neuropraxia following SCFH because of the possible higher likelihood of longer surgery duration. Pediatric orthopaedic surgeons should be prepared for more difficult and perhaps complicated fracture reduction.

## Limitations

Several limitations in our study should be taken into consideration. The nature of the study is retrospective and sample size is relatively small. Furthermore, lack of explicit surgical notes renders the recognition of specific reasons for surgeries duration difficult.

## Conclusion

Preoperative neuropraxia, severity of the fracture, and flexion-type fracture convey a potential longer duration of surgery in pediatric supracondylar fracture.
